# Effect of broiler genetics, age, and gender on performance and blood chemistry

**DOI:** 10.1016/j.heliyon.2020.e04400

**Published:** 2020-07-11

**Authors:** M.L. Livingston, A.J. Cowieson, R. Crespo, V. Hoang, B. Nogal, M. Browning, K.A. Livingston

**Affiliations:** a DSM Nutritional Products, 45 Waterview blvd. Parcipany, NJ, USA; b DSM Nutritional Products, Wurmisweg 576, 4303 Kaiseraugst, Switzerland; c Department of Population and Health Pathobiology, College of Veterinary Medicine, NC State University, Raleigh, NC 27607, USA; d InsideTracker, 101 Main Street, Cambridge, MA, USA; e Prestage Department of Poultry Science, NC State University, Raleigh, NC 27695-7608, USA

**Keywords:** Broiler, Biomarkers, Blood physiology, Rapid point-of-care detection, Animal breeding, Animal product, Biochemistry, Animal physiology, Veterinary science

## Abstract

A total of 640 broilers were used to determine the effects of strain, sex, and age on hematology and blood chemistry using rapid detection devices. Day old chicks from two genetic lines of common fast-growing and high-yield broiler strains were sexed and allocated to 40 pens (16 birds per pen) containing either male or female and Ross or Cobb strains (n = 10). Venous blood was analyzed weekly using 2 broilers from each pen (n = 20) using the i-STAT® Alinity Handheld Clinical Analyzer, Zoetis Vetscan VS2, and iCheck™ Carotene devices at 14, 21, 28, 35, and 42 d, as well as growth performance. Post-mortem health tracking metrics were also recorded on 42 d. Broilers were deemed healthy based on posting data results and performed in accordance with industry standards with males presenting greater BW and reduced FCR than female broilers. Ross broilers displayed greater BW to 14 d with similar FCR compared with Cobb birds. Day of age had a highly significant impact on blood calcium, phosphorus, potassium, sodium, chloride, carotene, aspartate aminotransferase, creatine kinase, bile acids, uric acid, total protein, albumin, globulin, total carbon dioxide, hematocrit, and malondialdehyde. Male broilers had reduced blood sodium, chloride, carotene, uric acid, albumin, and increased total protein, glucose, and total carbon dioxide. Ross broilers had greater blood potassium, and sodium, as well as reduced uric acid, total protein, globulin, and malondialdehyde, compared with Cobb birds. These results demonstrated the effectiveness of point-of-care devices in measuring blood chemistry and hematology in modern broilers. These data can be utilized to determine normal healthy blood ranges in these types of broilers when accounting for strain, sex, and age.

## 1 Introduction

Hematological and blood biochemical diagnosis of disease in human and veterinary medicine within individuals or a population is well established. The ability to compare individual data to a known healthy population mean can be used to identify outliers and diagnose specific disorders (Lindholm and Altimiras, 2016). The modern broiler industry lends itself well to this type of diagnosis as the individuals within this population are from well characterized genetic strain, diets, and environmental factors. While historic values have been determined accurately by validated bench top chemistry and analytical measures, those methods can be quite time consuming and costly. Technological advances in analytical devices have brought smaller, faster, and potentially portable methods for determining these values.

Hematological and biochemical parameters have been studied in avian species for many decades. Ross et al. (1976) utilized a variety of bench top analytical procedures to determine 14 different “comparison values” for 21 broiler flocks comprising over 900 samples at 42 d of age (Ross et al., 1976). While Meluzzi et al. (1992) examined nine similar blood markers at varying age, sex, strain, and seasons using analogous methods, and a similar study was conducted by Talebi et al. (2005) using hematological profiling. While other studies have focused on hematological and biochemical reference ranges only with regard to a specific time, strain, region, or sex (Adeleye et al., 2018; Al-Nedawi, 2018; Nanbol et al., 2016), others have studied the effects of various treatments on hematology including feeding allopurinol to reduce uric acid levels (Klandorf et al., 2001) or the effect of heat stress on similar blood biomarkers (Wang et al., 2018). Regardless of the objective, these hematological profiling experiments have occurred with increasing frequency.

Recently the use of more rapid detection methods known as Point-of-Care (POC) devices, which may include the i-STAT® Handheld Clinical Analyzer, Zoetis Vetscan VS2, and iCheck™ Carotene, have been utilized in animal care and research (Hoppes et al., 2015; Lindholm and Altimiras, 2016; Martin et al., 2010; Raila et al., 2017). These devices are portable bench top or handheld analytical devices used for rapid multi variable analysis of blood chemistry, gas, mineral, and other hematological factors. Moreover, these devices are beginning to be used to correlate various abnormalities in broilers including animal health, intestinal markers, and correlations to other disease (Celi et al., 2019; Lindholm and Altimiras, 2016; M L Livingston et al., 2019; Martin et al., 2010; Stinefelt et al., 2005).

It has been established that between flock variance of hematological comparison values has a significantly greater variance than within flock variance (Ross et al., 1976). Therefore, the objective of this study was to determine reference ranges for the i-STAT® Handheld Clinical Analyzer, Zoetis Vetscan VS2, and iCheck™ Carotene over the typical growth phases for similar Ross and Cobb modern fast-growing high yielding broilers of differing sexes raised concurrently. This study was designed to determine the blood chemistry and hematology expected values and ranges at weekly intervals of both Ross and Cobb, male and female broilers; as well as their final health status.

## 2 Materials and methods

### 2.1 Facilities and rearing

This trial was conducted between the months of February and April 2019. All live animal procedures used in this study were reviewed and approved by the Institutional Animal Care and Use Committee, North Carolina State University. Ross 708 and Cobb 500 chicks were sourced form a local commercial hatchery, vent-sex sorted, individually neck tagged, and allocated into 40 pens according to sex and strain to create a 2 × 2 factorial arrangement with 10 pens per treatment group. Pens were of uniform size (1.2 m × 1.2 m; 1.82 m^2^) with 16 chicks per pen in a closed, tunnel ventilated house. Each pen was supplied with one bell water drinker, one tube feeders, and bedded with fresh pine shavings (15 cm deep). All broiler chicks were assigned to the same corn-soy based diet in starter, grower, and finisher phases (Table 1).

**Table 1:** Composition of broiler starter, grower, and finisher diets.

Ingredient	Starter^4^	Grower^5^	Finisher^6^
	(%)		
Corn	57.65	64.50	66.53
Soybean meal (48% CP)	32.02	24.29	21.55
Poultry by-product meal	5.00	5.86	5.22
Poultry fat	2.00	2.51	3.99
Dicalcium phosphate (18.5% P)	1.24	0.83	0.67
Limestone	0.61	0.73	0.66
Salt	0.50	0.50	0.50
Choline chloride (60%)	0.20	0.20	0.20
Vitamin premix^1^	0.05	0.05	0.05
Mineral premix^2^	0.20	0.20	0.20
Selenium premix^3^	0.05	0.05	0.05
DL-Methionine	0.23	0.17	0.11
L-Lysine	0.14	0.20	0.13
L-Threonine	0.11	0.09	0.14
Total	100.00	100.00	100.00
Calculated nutrient content			
Crude protein	23.00	20.00	18.50
Calcium	0.90	0.80	0.70
Available phosphorus	0.45	0.40	0.35
Potassium	0.88	0.76	0.72
Total lysine	1.31	1.14	1.00
Total methionine	0.59	0.49	0.42
Total threonine	0.88	0.76	0.76
Total methionine + cysteine	0.95	0.81	0.72
Sodium	0.22	0.22	0.22
Chloride	0.23	0.23	0.23
Metabolizable energy (kcal/g)	2,935	3,050	3,150

1 Vitamin premix supplied the following per kg of diet: 13,200 IU vitamin A, 4,000 IU vitamin D_3_, 33 IU vitamin aE, 0.02 mg vitamin B_12_, 0.13 mg biotin, 2 mg menadione (K_3_), 2 mg thiamine, 6.6 mg riboflavin, 11 mg d-pantothenic acid, 4 mg vitamin B_6_, 55 mg niacin, and 1.1 mg folic acid.

2 Mineral premix supplied the following per kg of diet: manganese, 120 mg; zinc, 120 mg; iron, 80 mg; copper, 10 mg; iodine, 2.5 mg; and cobalt, 1 mg.

3 Selenium premix provided 0.2 mg Se (as Na_2_SeO_3_) per kg of diet.

4 Starter diet was fed to approximately 14 d of age, 910 g per bird.

5 Grower diet was fed from approximately 15 to 35 d of age, 2,750 g per bird.

6 Finisher diet was fed from approximately 36 to end of experiment.

### 2.2 Live performance and blood physiology

Broiler BW and feed consumption were recorded at 1, 7, 14, 21, 28, 35, and 42 d of age and FCR calculated. At 14, 21, 28, 35, and 42 d of age two broilers per pen were selected for venous blood analysis. Approximately 4–5 mL of venous blood was initially drawn and allocated into three blood collection tubules as 0.5 ml Heparinized, 1 + ml EDTA treated, and 3 ml serum collection tubules. A systematic approach to catching broilers and drawing of blood was developed to assure uniform collection and analysis of samples.

Heparinized blood (approximately 0.2 ml) was analyzed in the i-Stat® Alinity v handheld blood analyzer fitted with a Chem8+ cartridge (Abbott Point of Care Inc., Princeton, NJ), which measured hematocrit (Hct) hemoglobin (Hb), blood urea nitrogen (BUN), creatinine (Crea), ionized calcium (^i^Ca), glucose (^i^Glu), chloride (^i^Cl), sodium (^i^Na), potassium (^i^K), total carbon dioxide (TCO2), anion gap (^i^AnGap).

Remaining heparinized blood (0.1 ml) was analyzed in the Vetscan® VS2 Chemistry Analyzer (Zoetis, Inc) using the Avian/Reptilian Profile Plus cartridge (Zoetis, Inc). This resulted in aspartate aminotransferase (AST), creatine kinase (CK), uric acid (UA), glucose (^v^GLU), calcium (^v^Ca), phosphorus (^v^P), total protein (TP), albumin (aLB), albumin/globulin (GLOB), potassium (^v^K) and sodium (^v^Na).

Precisely 1.0 ml of EDTA blood was mixed with 0.20 ml cellular fixant (Transfix®, MBL International), stored and shipped on wet ice to Cayman Analytical Laboratories (Ann Arbor, MI) for Heterophile to Lymphocyte ratio analysis using Flow Cytometry as described by Lentfer et al. (2015) and Bílková et al. (2017) (Bílková et al., 2017; Lentfer et al., 2015).

Whole blood (3 ml) was spun and serum removed and stored on wet ice. 0.40 ml serum was used for total carotenoids (mg/kg) using the iCheck® carotene photometer device and test kit (BioAnalyt GmbH, Potsdam, Germany) as described by Kawashima et al. (2010) (Kawashima et al., 2010). Remaining serum was frozen on dry ice and shipped to Cayman Analytical Laboratories (Ann Arbor, MI) for TBARS analysis.

### 2.3 Post-mortem animal health evaluations

Broilers subject to 42 d blood draw were immediately euthanized and evaluated for bursal meter score (BSM, 1–9), tibial dyschondroplasia (TD, 0–3), and presence or absence of burned feed (BF), femoral head necrosis (FH), and thymus atrophy (Thymus) (Saif et al., 2008a, 2008b). Carcasses rested for 4 h to allow resolution of rigor then evaluated for white striping (WS) and wooden breast (WB) myopathic scores on a 1–4 point scale (Matthew L Livingston et al., 2019).

### 2.4 Statistical analysis

Data were analyzed using JMP Pro 14 (“SAS Institute,” 2009). Live performance and blood physiology data were analyzed as a two-way ANOVA by day with means separated with Tukey's adjustment for multiple comparisons test (P < 0.05). Pen was considered the individual experimental unit for live performance parameters, while individual blood draw was considered the experimental unit (n = 20) for blood physiology parameters. Analysis of 42 d health status scores were analyzed as independent variables with similar dependent variables as above and the GLM procedure of SAS. The PROC GLIMMIX procedure of JMP Pro 14 (“SAS Institute,” 2009) was used to analyze BF, FH, Thymus, data shown as % detectable.

Blood physiology data were analyzed as a three-way ANOVA with means separates with Tukey's adjustment for multiple comparisons test (P < 0.05) using strain, sex, and day of age as independent variables; data are presented as main effect means for brevity, with interaction model p-values indicated and described fully in text. Finally, linear and quadratic regression models were used to explore the influence of biomarker over age, data presented as regression coefficients.

## 3 Results and discussion

### 3.1 Live performance

Effects of strain and sex on BW, FC, and FCR can be found in Tables 2, 3, and 4; respectively. Ross chicks had greater 0 d BW when compared to Cobb chicks (P < 0.01). Greater BW (P < 0.02) was observed in Ross males from 0 – 14 d of age when compared to Cobb males, while male broiler BW exceeded that of females in both strains from 14 - 42 d (P < 0.01). Ross male BW exceeded that of Cobb males and all females at 14 d (P < 0.05), while Cobb males exceeded all female BW at 21 d (P < 0.05), which indicated an earlier onset of growth or possibly maturity in Ross males when compared to Cobb males. The greater BW of Ross birds at d of hatch could be attributed to differences in breeder flock age, or genetics (Nangsuay et al., 2017) and this initial difference could also have explained the greater BW from d 0 to 14.

**Table 2:** Effect of strain (Ross or Cobb) and Sex (Male or Female) on weekly Body Weights (kg).

Strain^2^	Sex	Weekly Body Weight^1^						
		0 d	7 d	14 d	21 d	28 d	35 d	42 d
		(kg/broiler)						
Ross		0.038	0.16	0.58	1.25	1.73	2.44	3.24
Cobb		0.035	0.15	0.56	1.23	1.70	2.42	3.20
	Male	0.037	0.16	0.58	1.29	1.83	2.63	3.52
	Female	0.036	0.16	0.56	1.19	1.61	2.23	2.91
SEM^3^		0.000	0.002	0.004	0.012	0.015	0.016	0.020
Ross Male		0.038	0.16	0.60^a^	1.32^a^	1.85	2.66	3.55
Ross Female		0.037	0.16	0.56^b^	1.18^c^	1.61	2.22	2.92
Cobb Male		0.035	0.15	0.57^b^	1.26^b^	1.80	2.61	3.50
Cobb Female		0.035	0.15	0.55^b^	1.20^c^	1.61	2.23	2.90
SEM^3^		0.000	0.002	0.006	0.016	0.021	0.023	0.028
		(Probability > F)						

Strain		<0.01	<0.01	<0.01	0.21	0.19	0.36	0.13
Sex		0.38	0.73	<0.01	<0.01	<0.01	<0.01	<0.01
Strain x Sex		0.10	0.30	0.02	0.02	0.24	0.16	0.60

Means within a column of 3 or more independent variables lacking a common superscript differ significantly (P < 0.05^a,b^).

1 Main and interaction effect means calculated using total pen weight/number of birds per pen = BW (kg).

2 Ross 708 and Cobb 500.

3 SEM = Standard error of mean, main effect means of strain and sex calculated using n = 20.

**Table 3:** Effect of strain (Ross or Cobb) and Sex (Male or Female) on weekly feed consumption (FC = g/bird/day).

Strain^2^	Sex	Weekly Feed Consumption^1^					
		0–7 d	7–14 d	14–21 d	21–28 d	28–35 d	35–42 d
		(g)					
Ross		22.64	68.93	113.52	140.33	188.86	231.52
Cobb		21.17	67.87	114.79	139.41	187.71	231.34
	Male	21.83	69.72	118.90	149.28	202.15	244.24
	Female	21.98	67.08	109.41	130.46	174.42	218.62
SEM^3^		0.34	0.62	0.92	0.98	1.15	2.47
Ross Male		22.50	71.10	120.08^A^	150.99	204.01	245.77
Ross Female		22.79	66.76	106.96^C^	129.66	173.72	217.28
Cobb Male		21.16	68.34	117.72^A^	147.57	200.29	242.72
Cobb Female		21.17	67.41	111.86^B^	131.24	175.13	219.96
SEM^3^		0.48	0.88	1.31	1.38	1.63	3.49
		(Probability > F)					

Strain		0.004	0.237	0.336	0.511	0.482	0.957
Sex		0.755	0.005	<0.001	<0.001	<0.001	<0.001
Strain x Sex		0.783	0.059	0.009	0.080	0.125	0.482

^ab^ Means within a column of 3 or more independent variables lacking a common superscript differ significantly (P < 0.05).

1 Main and interaction effect means calculated using total pen feed consumption (g)/number of birds/days = FC (g/bird/day).

2 Ross 708 and Cobb 500.

3 SEM = Standard error of mean, main effect means of strain and sex calculated using n = 20.

**Table 4:** Effect of strain (Ross Cobb) and Sex (Male or Female) on weekly feed conversion ratio (FCR = g:g).

Strain^2^	Sex	Weekly FCR^1^					
		0–7 d	0–14 d	0–21 d	0–28 d	0–35 d	0–42 d
Ross		1.272	1.229	1.312	1.385	1.445	1.775
Cobb		1.267	1.242	1.330	1.398	1.447	1.801
	Male	1.262	1.223	1.305	1.367	1.406	1.711
	Female	1.277	1.248	1.337	1.416	1.486	1.866
SEM^3^		0.015	0.010	0.007	0.008	0.007	0.012
Ross Male		1.251	1.211	1.296	1.364	1.407	1.704
Ross Female		1.293	1.247	1.328	1.406	1.484	1.847
Cobb Male		1.273	1.234	1.315	1.370	1.405	1.717
Cobb Female		1.261	1.249	1.346	1.426	1.489	1.885
SEM^3^		0.022	0.014	0.010	0.012	0.010	0.017
		(Probability > F)					

Strain		0.835	0.160	0.009	0.400	0.154	0.073
Sex		0.513	0.068	0.002	0.001	0.001	0.009
Strain x Sex		0.222	0.694	0.117	0.844	0.475	0.646

^ab^ Means within a column of 3 or more independent variables lacking a common superscript differ significantly (P < 0.05).

1 Main and interaction effect means calculated using total pen feed consumption (g)/growth (g) = FCR (g:g).

2 Ross 708 and Cobb 500.

3 SEM = Standard error of mean, main effect means of strain and sex calculated using n = 20.

Feed consumption was higher in Ross broilers from 0-7 d (P < 0.01) when compared to Cobb (Table 3). However, similar feed intake was measured between strain from 7-42 d. Male broilers consumed a greater amount of feed when compared to females from 7-42 d (P < 0.01), as was expected. However, Ross females consumed less feed than cobb females from 14-21 d, which resulted in a significant interaction effect (P < 0.01). A similar interaction trend was observed prior and subsequent to this period beginning at 7–14 and ending 21–28 d (P < 0.10).

Male broilers displayed reduced FCR from 14–42 d of age when compared to females (P < 0.01), as was expected (Table 4). When compared to Cobb, Ross broilers had superior FCR from 0-21 d (P < 0.01). Males of both strains outperformed females as evidenced by greater BW beginning at 14 d and improved FCR at 21d, as was expected. However, Ross broilers tended toward lower FCR from 0-42 d, when compared to Cobb broilers (0.025 g:g; P < 0.08).

Broilers in this study performed in accordance with breeder guidelines. Male broilers outperformed female broilers as indicated by increased FC resulting in greater BW and reduced FCR. This effect is well established in the literature (Brewer et al., 2012; Havenstein et al., 1994; Zuidhof et al., 2014). While Cobb females consumed more feed in the grower phase, this effect did not translate into improved BW or FCR. The limited effect of performance parameters between male Ross and male Cobb can most likely be attributed to a single diet formulated to meet or exceed NCR requirements (NRC, 1994), as opposed to the precision nutritional strain and sex recommendations.

### 3.2 Blood physiology

A total of 400 individual venous blood draws were obtained from male and female broilers of two genetic strains at 5 weekly intervals (14–42 d). The effect of age on blood chemistry and hematology was strongly apparent (Tables 5, 6, and 7). Except for Glu, ^v^Ca, and ^i^Cl, all blood biomarkers had a highly significant linear response to age. Those with linear responses also responded quadratically with the exception of ^v^Na, GLOB, and H:L. Genetic line contributed to significant differences in ^v^K, ^i^Na, UA, TP, GlOB and MDA, while sex impacted ^i^Na, ^i^Cl, carotene, UA, TP, ALB, ^v^GLU, ^i^GLU, TCO2, HCT and MDA (P < 0.05).

**Table 5:** Effects of strain (Cobb or Ross), sex (Male or Female) and age on blood mineral composition of broiler chickens fed a nutritionally adequate corn/soy-based diet.

Strain	Sex	Age	^v^Ca	^v^PHOS	^v^K	^v^Na	^i^Na	^i^K	^i^Cl	^i^Ca	^i^AnGap
			(mg/dL)		(mmol/L)						
Cobb			11.52	7.27	7.36	148.1	143.6	4.93	108.3	1.36	16.2
Ross			11.48	7.32	8.12	149.2	144.1	4.96	108.6	1.35	16.4
P <			0.171	0.721	<0.001	0.199	0.011	0.484	0.062	0.386	0.557
	Male		11.48	7.35	7.78	148.3	143.6	5.01	107.6	1.35	16.4
	Female		11.53	7.23	7.70	149.0	144.1	4.88	109.1	1.36	16.3
	P <		0.802	0.380	0.409	0.807	0.015	0.140	0.009	0.071	0.631
		14 d	11.43^bc^	6.11^c^	6.69^cd^	146.1^b^	140.9^c^	5.55^a^	110.6^a^	1.26^c^	12.9^b^
		21 d	11.97^a^	7.45^ab^	7.14^bc^	149.6^a^	144.0^b^	5.11^b^	108.3^ab^	1.39^a^	17.2^a^
		28 d	11.26^bc^	7.91^a^	8.17^ab^	148.1^b^	143.5^b^	4.63^cd^	107.0^b^	1.33^b^	16.9^a^
		35 d	11.72^ab^	7.26^b^	9.07^a^	149.5^a^	145.5^a^	4.52^d^	106.7^b^	1.39^a^	16.8a
		42 d	11.13^c^	7.74^ab^	7.64^d^	149.8^a^	145.3^a^	4.94^bc^	108.9^ab^	1.39^a^	17.8^a^
		P <	<0.001	<0.001	<0.001	0.022	<0.001	<0.001	<0.001	<0.001	<0.001
		Linear P <	0.032	<0.001	<0.001	0.012	<0.001	<0.001	0.021	<0.001	<0.001
		Quadratic P <	0.027	<0.001	<0.001	0.421	<0.001	<0.001	<0.001	0.010	<0.001
Model Pooled SEM^1^			0.25	0.32	0.48	1.9	0.5	0.19	1.3	0.02	0.5

**Interaction Terms**			(Probability > F)								

Strain∗Age			0.691	0.923	0.005	0.138	0.319	0.673	0.248	0.275	0.461
Sex∗Age			0.026	0.778	0.487	0.487	0.967	0.017	0.218	0.244	0.027
Strain∗Sex			0.001	0.080	0.773	0.886	0.398	0.804	0.302	0.064	0.711
Strain∗Age∗Sex			0.002	0.023	0.173	0.063	0.131	0.519	0.566	0.655	0.143

^ab^ Means within a column of 3 or more independent variables lacking a common superscript differ significantly (P < 0.05).

1 SEM = Standard error of mean, main effect means of strain and sex calculated using n = 200, main effect means of age calculated using n = 80.

**Table 6:** Effects of strain (Cobb or Ross), sex (Male or Female) and age on broiler blood carotene, aspartate aminotransferase (AST), creatine kinase (CK), bile acids (BA), uric acid (UA), total protein (TP), albumin (ALB), globulin (GLOB).

Strain	Sex	Age	Carotene	AST	CK	BA	UA	TP	ALB	GLOB
			(mg/kg)	(U/L)		(μmol/L)	(mg/dL)	(g/L)		
Cobb			2.30	393.0	4359	11.37	6.3	3.06	2.33	0.73
Ross			2.30	392.3	4017	10.10	5.7	2.96	2.31	0.64
P <			0.982	0.751	0.570	0.243	0.003	<0.001	0.305	<0.001
	Male		2.17	386.7	4064	10.28	5.6	3.04	2.28	0.68
	Female		2.42	398.0	4313	11.19	6.4	2.97	2.35	0.69
	P <		<0.001	0.781	0.605	0.404	<0.001	0.011	0.002	0.928
		14 d	1.49^d^	193.7^d^	2401^b^	12.6^a^	6.2^b^	2.67^c^	2.20^b^	0.45^c^
		21 d	2.10^c^	225.7^d^	4908^a^	13.4^a^	6.2^b^	2.95^b^	2.36^a^	0.59^b^
		28 d	2.26^bc^	335.6^c^	5255^a^	11.4^ab^	6.3^b^	2.97^b^	2.31^a^	0.64^b^
		35 d	3.09^a^	494.5^b^	.	9.1^ab^	6.9^b^	3.25^a^	2.39^a^	0.87^a^
		42 d	2.55^b^	713.6^a^	.	7.2^b^	4.4^a^	3.21^a^	2.34^a^	0.87^a^
		P <	<0.001	<0.001	<0.001	<0.001	<0.001	<0.001	<0.001	<0.001
		Linear P <	<0.001	<0.001	<0.001	<0.001	<0.001	<0.001	<0.001	<0.001
		Quadratic P <	<0.001	<0.001	<0.001	<0.001	<0.001	<0.001	<0.001	0.436
Model Pooled SEM^1^			0.140	42.79	727	727	727	2.6	0.05	0.06

**Interaction Terms**			(Probability > F)							

Strain∗Age			0.283	0.702	0.505	0.625	0.219	0.012	0.571	0.103
Sex∗Age			0.058	0.685	0.052	0.068	0.002	0.261	0.016	0.507
Strain∗Sex			0.245	0.675	0.509	0.114	0.429	<0.001	0.050	0.048
Strain∗Age∗Sex			0.381	0.023	0.020	0.179	0.171	0.285	0.775	0.358

^ab^ Means within a column of 3 or more independent variables lacking a common superscript differ significantly (P < 0.05).

1 SEM = Standard error of mean, main effect means of strain and sex calculated using n = 200, main effect means of age calculated using n = 80.

**Table 7:** Effects of strain (Cobb or Ross), sex (Male or Female) and age on blood mineral composition of broiler chickens fed a nutritionally adequate corn/soy-based diet.

Strain	Sex	Age	^v^Glu	^i^Glu	TCO2	HCT	MDA	H	L	H:L
			(mg/dL)	(mmol/L)		(%)	(μM)			
Cobb			235	240	24.9	20.1	0.84	2175	8743	0.25
Ross			239	241	24.7	19.8	0.78	2421	9583	0.25
P <			0.543	0.614	0.164	0.410	0.047	0.10	0.02	0.918
	Male	239	243	25.1	19.5	0.81	2180	8711	0.25	
	Female	234	237	24.4	20.3	0.81	2416	9615	0.25	
	P <		0.033	0.008	0.006	0.052	0.883	0.11	0.010	0.910
		14 d	235^ab^	243	23.5	18.9	1.14	1978^b^	6924^d^	0.28^ab^
		21 d	240^ab^	239	24.5	19.0	1.12	2912^a^	9165^c^	0.32^a^
		28 d	242^a^	244	25.1	20.5	0.63	2777^a^	10720^b^	0.26^bc^
		35 d	238^ab^	240	26.3	21.6	0.48	1182^c^	6388^d^	0.15^d^
		42 d	231^b^	235	24.6	19.7	0.69	2640^a^	12620^a^	0.21^c^
		P <	0.039	0.082	<0.001	<0.001	<0.001	<0.001	<0.001	<0.001
		Linear P <	0.248	0.042	<0.001	0.005	<0.001	0.412	<0.001	<0.001
		Quadratic P <	0.003	0.252	<0.001	0.0121	<0.001	0.629	0.192	0.847
Model Pooled SEM^1^			6	5	0.6	0.9	0.07	332	774	0.03

**Interaction Terms**			(Probability > F)							

Strain∗Age			0.050	0.074	0.319	0.089	<0.001	0.215	0.550	0.391
Sex∗Age			0.314	0.323	0.024	0.102	0.776	0.361	0.582	0.641
Strain∗Sex			0.134	0.147	0.885	<0.001	0.198	0.332	0.843	0.654
Strain∗Age∗Sex			0.104	0.104	0.335	0.951	0.206	0.884	0.407	0.615

^ab^ Means within a column of 3 or more independent variables lacking a common superscript differ significantly (P < 0.05).

1 SEM = Standard error of mean, main effect means of strain and sex calculated using n = 200, main effect means of age calculated using n = 80.

Effects of broiler strain, sex, and age on blood mineral composition can be found in Table 5. All broiler blood minerals measured by both devices (^v^Ca, ^v^Phos, ^v^K, ^v^Na, ^i^Na, ^i^K, ^i^Cl, ^i^Ca, and ^i^AnGap) resulted in a highly significant age response (P < 0.001, ^v^Na = 0.02); as well as a linear effect (P < 0.05); and with the exception of ^v^Na, a quadratic effect (P < 0.05).

Ross ^v^K was greater compared to Cobb broilers at 35 d, while a quadratic effect of age on ^v^K (P < 0.001) was also observed. This resulted in a strain∗age interaction (P < 0.005). Moreover, ^i^K responded quadratically to age (P < .001) with an increase from 35 to 42 d observed in females; however, that response was only observed in females creating a Sex∗Age interaction (P < 0.017). Finally, Ross female and Cobb male blood ^v^Phos was greater at 28 d when compared to Ross male and Cobb female broilers at the same time period, resulting in a strain∗sex∗age interaction (P < 0.01). Interaction effects of strain∗sex∗age on blood mineral values are not shown for brevity; however, at 14 d ^i^Na and ^i^Cl levels were reduced (P < 0.05) in Cobb males, while females of both strains had increased ^i^Ca at 21 d (P < 0.01).

Effects of strain, sex, and age on broiler blood protein, acids, and carotene composition can be found in Table 6. A significant age effect (P < 0.001) was found for carotene, AST, CK, BA, UA, TP, ALP, GLOB. These effects were confirmed by linear (P < 0.001), and apart from GLOB, quadratic (P < 0.001) regressions. Broiler CK levels were not recognized by the i-Stat® Alinity at 35 and 42 d of age due to greater than detectable levels. Males displayed greater carotene, UA, ALB, and reduced TP (P < 0.01) when compared to female broilers. Cobb broilers had higher UA, TP, and GLOB when compared to Ross broilers (P < 0.01).

Total protein (TP) was greater in Cobb females compared with other broilers, resulting in a strain∗sex interaction (P < 0.001). Cobb broilers had elevated blood TP (<0.01) at earlier ages when compared to Ross broilers, which resulted in a strain∗age interaction. Interaction effects of strain∗sex∗age on broiler blood protein, acids, and carotene are not shown for brevity; however, GLOB was greater in Cobb broilers at 14, 21, and 35 d (P < 0.05) and tended to be greater at 28 d when compared to Ross broilers. Cobb also displayed greater TP and UA at 21, 28 and 35 d (P < 0.01) when compared to Ross broilers, while males of both strains had reduced UA at 35 and 42 d (P < 0.01). Finally, BWA of females was greater (P < 0.01) at 42 d with a trend at 35 d when compared to male broilers.

Effects of broiler strain, sex, and age on hematology results including ^v^Glu, ^i^Glu, TCO2, HCT, MDA, and H:L ratios can be found in Table 7. A significant age effect was observed in TCO2, HCT, MDA, and H:L ratio (P < 0.01), and ^v^Glu (P < 0.05). A significant linear effect was present in TCO2, HCT, MDA, and H:L ratio (P < 0.01), and a quadratic effect in TCO2, HCT, and MDA (P < 0.01). Male broilers had greater ^v^Glu, ^i^Glu, and TCO2 compared to female broilers (P < 0.05), while Cobb broilers displayed greater MDA levels compared to Ross broilers (P < 0.05).

Changes in blood chemistry and hematology in relationship to broiler age, sex, and strain have been reported previously. For example, Meluzzi et al. (1992) reported an increase in broiler plasma phosphorus, aspartate aminotransferase, and total protein as birds aged. While Kawasaki et al. (2018) reported an increase in CK and AST levels as broilers aged. The results of ^v^Phos, AST, and TP in the present study (Tables 5 and 6) also agreed with the published findings, which resulted in a linear increase as broilers aged (Kawasaki et al., 2018; Meluzzi et al., 1992). An increase in AST alone could be an indicator of hepatocellular injury (Hoffmann and Solter, 2008). However, given the increased CK values observed in the present study, even those that were eliminated from statistical observations (Table 6) due to greater than detectable levels, it is much more likely that the AST values are attributed to muscle turnover and increased growth rate rather than that of hepatocellular damage. The increase in both AST and CK has been reported in multiple studies investigating increased muscle damage, turnover, and regrowth (Abaxis Inc., 2007; Ramirez et al., 1973; Tanzer and Gilvarg, 1959). It should be noted however, that the increased CK in the serum may have a direct impact on the detected ^v^K values. The Vetscan® VS2 utilizes a coupled pyruvate kinase/lactate dehydrogenase assay to measure ^v^K values, which will result in falsely elevated levels of ^v^K when significantly greater levels of CK are present. Uniquely, ^i^K values decreased significantly over time and only increased from 35 to 42 d.

It has been reported that higher serum protein and lower globulin values were observed in more efficient (lower FCR) birds due to superior utilization of nutritional protein (Adeleye et al., 2018). These results agreed with the present study where lower FCR and greater BW of Ross male broilers was associated with lower GLOB values, when compared to Cobb broilers; and greater TP values when compared to female broilers. This suggests that broilers in the present study utilized nutritional protein more efficiently, which manifested as decreased FCR.

Meluzzi et al. (1992) reported no significant differences in calcium or phosphorus values between either strain or sex, which also agreed with the ^v^Ca, ^i^Ca, and ^v^PHOS findings of these broiler groups in the present study (Table 5). Blood ^v^Na and ^i^Na increased with age in the present study, agreeing with the observations of Nanbol et al. (2016), where blood Na was reported to increase in broilers as they aged from 4 to 8 weeks. The significant main effect increases of ^i^Na associated with both Ross strain and female sex can be attributed primarily to a significant decrease of ^i^Na in Cobb males at 14 d, and significant decrease of ^i^Na in all Cobb broilers at 21 d (interaction data not shown for brevity). A similar trend was observed in ^i^Cl for the same treatment groups at 14 and 21d. These data may suggest an association with decreased performance as noted by significantly reduced BW at similar time periods.

The results of the present study observed a reduction in H:L ratio with age (Table 7). A similar negative correlation of H:L to broiler age was has previously been reported (Talebi et al., 2005). In the present study this can be attributed to a greater increase in lymphocytes than heterophiles, although both counts increased with age. Previous research has demonstrated a relationship between H:L ratio, broiler stress, and BW (Weimer et al., 2018). Data in this current trial agreed with those findings, which resulted in a negative linear response with H:L ratio and BW.

Plasma carotene concentration has been associated with increased nutritional absorption (Celi et al., 2019). The increased feed consumption relative to BW in female broilers could explain the result of increased blood carotene by increased consumption of feed. This may explain why carotene values in the current trial increased as broilers aged from 14 to 35 d, and then decreased from 35 to 42 d, while females exhibited significantly greater carotene throughout the study (Table 6). Previous research has determined that dietary canthaxanthin did not affect other measured blood analytes; however, broiler plasma CK values have been reported to decrease when canthaxanthin was consumed in greater amounts during aflatoxin exposure (Okotie-Eboh et al., 1997). In the present study carotene was not correlated to other blood analytes, which may indicate a lack of stress associated with aflatoxin exposure.

In the present study broiler plasma UA held relatively steady from 14 – 35 d, however dropped significantly at 42 d (Table 6). Cobb and female broilers resulted in greater UA concentrations than Ross and male broilers throughout the study. The UA concentration of avian species is unique to the animal kingdom. While UA has been found to protect cellular membranes and DNA from reactive oxygen species (Stinefelt et al., 2005), its large concentration has given broilers (and other avian) improved longevity compared to mammalian species through its free radical scavenging and decreased oxidative stress capabilities (Ku and Sohal, 1993; Lindstedt and Calder, 1976).

Being a highly reactive compound, MDA is associated with oxidative stress and is generated from lipid peroxidation resulting in an increase of free radicals (Campbell, 2015). In the present study MDA was greater in Cobb broilers at an earlier age of 14–21 d, however was not significant after 28 d (data not shown for brevity). This increase in MDA may be associated with a reduction in BW during the same time period. Broiler MDA was not correlated to UA concentrations in this study.

In the present study broiler blood glucose levels (^i^Glu, ^v^Glu) were highly correlated (r^2^ = 0.97, P < 0.01), and although their values differed among age groups they showed minimal linear or quadratic response to age (Table 7). However, when looking at the full model and interaction effects of strain∗sex∗age (data not shown for brevity) the only significant differences within ^i^Glu or ^v^Glu occur at 35 d. Ross male broilers exhibited a 10% increase in blood glucose levels at 35d when compared to other broilers (P < 0.01), while cobb males show a similar trend with a 6% increase in blood ^i^Glu or ^v^Glu at 42 d (P = 0.10).

### 3.3 Post-mortem animal health evaluations

Effects of broiler strain, sex, and age on post-mortem health conditions and breast myopathies can be found in Table 8. Health conditions of the broilers appeared to be normal with no signs of chronic or acute disease except for the muscle myopathies observed. Male broilers were more susceptible to increased WS and WB scores, while Ross strain was more susceptible to greater WB scores (P < 0.01). These data agreed with the previously published literature (Kuttappan et al., 2013; Matthew L Livingston et al., 2019; Trocino et al., 2015) where males exhibited greater WB and WS scores and occurred at increased percentages when compared to females.

**Table 8:** Effect of strain (Cobb or Ross), sex (Male or Female) on white striping (WS), wooden breast (WB), bursal meter score (BSM), tibial dyschondroplasia (TD), and percentage of positive broilers with burned feed (BF), femoral head necrosis (FH), and thymus at 42 d of age.

Strain	Sex	WS	WB	BSM	TD	BF∗	FH∗	Thymus∗
		(1–4)		(1–9)	(0–3)	(% +)	(% +)	(% +)
Cobb		1.87	2.25	1.28	0.10	2.6	35.9	12.8
Ross		2.00	2.80	1.18	0.15	7.5	32.5	7.5
P <		0.387	<0.001	0.327	0.546	0.321	0.715	0.431
	Male	2.12	2.73	1.10	0.18	5.0	40.0	5.0
	Female	1.75	2.33	1.36	0.08	5.1	28.2	15.4
	P <	0.011	0.013	0.022	0.245	1.0	0.286	0.131
Cobb	Female	1.65	2.15	1.37	0.05	0.0	36.8	15.8
Cobb	Male	2.10	2.35	1.20	0.15	5.0	35.0	10.0
Ross	Female	1.85	2.50	1.35	0.10	10.0	45.0	0.0
Ross	Male	2.15	3.10	1.00	0.20	5.0	20.0	15.0
	P <	0.604	0.209	0.415	0.987	0.320	0.216	0.501
Model Pooled SEM^1^		0.14	0.16	0.11	0.08	.	.	.

^ab^Means within a column of 3 or more independent variables lacking a common superscript differ significantly (P < 0.05, ∗Prob > ChiSq).

1 SEM = Standard error of mean, main effect means of strain and sex calculated using n = 40, interaction effect means calculated using n = 20.

## 4 Conclusions

The present study investigated the impact of strain and sex over time on multiple POC devices, which measured several blood biomarkers of two modern, fast-growing broiler strains fed the same diet. While strain influenced only ^v^K, ^i^Na, UA, TP, GLOB, and MDA; broiler sex contributed to changes in ^i^Na, ^i^Cl, carotene, UA, TP, ALB, ^i^GLU, ^v^GLU, TCO2, HCT, and MDA. A persistent impact of age was noted in all biomarkers measured and this should be allowed within the model and the experimental design of future studies. It was notable that total protein, albumin, and globulin biomarkers may be an indicator of performance when comparing between strains, and this may translate into performance markers between flocks.

The use of these POC devices to determine normal blood chemistry and hematology can be useful and rapid. Care must be utilized when interpreting the results of the data as many of these parameters are dependent on age, sex, and strain. If these devices are used to determine health status of broilers, these contributary factors should be used to narrow the expected “baseline” results. Further research is needed to determine the effect of known health status on broilers, however these findings should allow for the determination of common mean values for the blood chemistry and hematology known for these POC devices.

## Declarations

### Author contribution statement

M. L. Livingston: Conceived and designed the experiments; Performed the experiments; Analyzed and interpreted the data; Wrote the paper.

A. J. Cowieson: Conceived and designed the experiments; Analyzed and interpreted the data; Wrote the paper.

R. Crespo: Performed the experiments; Contributed analysis tools or data; Wrote the paper.

V. Hoang, B. Nogal: Analyzed and interpreted the data; Contributed analysis tools or data.

M. Browning: Performed the experiments; Contributed analysis tools or data.

K. A. Livingston: Conceived and designed the experiments; Performed the experiments; Wrote the paper.

### Funding statement

This work was supported by DSM Nutritional Products, Kaiseraugst, Switzerland.

### Competing interest statement

The authors declare no conflict of interest.

### Additional information

No additional information is available for this paper.
